# Measuring the Bandgap of Ambipolar 2D Semiconductors using Multilayer Graphene Contact

**DOI:** 10.1002/smsc.202200075

**Published:** 2022-12-22

**Authors:** Sam Park, Sungjae Hong, June Yeong Lim, Sanghyuck Yu, Jungcheol Kim, Hyeonsik Cheong, Seongil Im

**Affiliations:** ^1^ vdWMRC Department of Physics Yonsei University 50 Yonsei-ro Seodaemun-gu Seoul 03722 Korea; ^2^ Department of Physics Sogang University 35 Baekbeom-ro Mapo-gu Seoul 04107 Korea

**Keywords:** ambipolar field-effect transistors, bandgap approximation, black phosphorus, multi-LG, TMD

## Abstract

The bandgaps of monolayers and few layers in 2D semiconductors are usually measured by optical probing such as photoluminescence (PL). However, if their exfoliated thickness is as large as a few nanometers (multilayer over ≈5 L), PL measurements become less effective and inaccurate because the optical transition of a 2D semiconductor often changes from direct to indirect mode. Herein, a simple method to approximately estimate the bandgap of multilayer 2D van der Waals (vdW) semiconductors is introduced; that is utilizing a field‐effect transistor (FET) as a platform. Multilayer graphene (multi‐LG) contact for multilayer van der Waals channels in the FET is used, because multi‐LG contact would secure ambipolar behavior and somewhat enable Schottky barrier modulation in contact with vdW channels. As a result, the bandgaps of multilayer transition‐metal dichalcogenides (TMDs) and black phosphorus in unknown thicknesses are approximated through measuring the temperature‐dependent transfer curve characteristics. The bandgaps are confirmed with photoelectric responsivity measurements, which evidences the validity of the multi‐LG‐induced approximation.

## Graphical abstract

A novel bandgap estimation technique utilizing a multilayer graphene (multi‐LG) source/drain contact field‐effect transistor (FET) as a platform is introduced. The gate‐voltage tunability of Schottky barrier at the 2D material/multi‐LG junction makes it possible to extract the half‐bandgap value from the temperature‐dependent transfer characteristics of the FET. The bandgaps of multilayer transition‐metal dichalcogenides (TMDs) and black phosphorus are approximated with the technique.

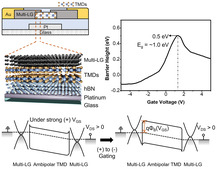

## 1 Introduction

The 2D van der Waals semiconductors have been extensively studied for the last decade, regarded as one kind of the most important breakthrough materials toward future device technologies.^[^ ^1^, ^2^, ^3^, ^4^, ^5^, ^6^, ^7^, ^8^, ^9^, ^10^, ^11^ ^]^ Many of transition‐metal dichalcogenides (TMDs) and black phosphorus (BP) would be the representatives of the 2D van der Waals (vdW) semiconductors, which are mechanically exfoliated to be monolayer (1L), bilayer (2L), and even multilayer.^[^ ^12^, ^13^, ^14^, ^15^, ^16^, ^17^, ^18^, ^19^, ^20^ ^]^ Bandgap of monolayer and few layer in 2D semiconductors has been reported, measured by optical probing such as photoluminescence (PL).^[^ ^20^, ^21^, ^22^, ^23^ ^]^ However, if their exfoliated thickness is as large as a few nanometers (multilayer over 5L), PL measurements become less effective and inaccurate because the optical transition of 2D semiconductor is changed from direct to indirect mode.^[^ ^24^, ^25^ ^]^ Along with the optical transition, PL peak is broadened in shape and its position is not well defined. At the moment, the energy bandgap also becomes smaller with the semiconductor thickness,^[^ ^25^, ^26^ ^]^ and optical absorption/reflection measurement of thick multilayer 2D semiconductor bandgap (*E*_g_) is not easy due to its own size limit in flake (≈ a few micrometers). Hence, density‐function theory (DFT)‐based calculations have been a main method to estimate the bandgap in general. Experimental methods beyond PL have also been attempted with such a variety of techniques as scanning tunneling microscopy, microprobe absorption/reflectance spectroscopy, and spectral photoelectric measurements in report.^[^ ^27^, ^28^, ^29^, ^30^, ^31^, ^32^, ^33^, ^34^, ^35^ ^]^ However, most of those techniques need specific equipment setups, which are often complex requiring extra skills. Here, we introduce a relatively simple method to measure the bandgap of multilayer 2D semiconductors; that is utilizing field effect transistor (FET) as a platform, which is but using multilayer graphene (multi‐LG, thin graphite) contact for source and drain (S/D). Our method is thus for transport gap, which is more practical and closer to authentic *E*_g_ than optical gap. We have fabricated multilayer 2D channel FETs with multi‐LG contact and top passivation for the present study, because multi‐LG would secure ambipolar behavior and enable Schottky contact barrier modulation in a degree with the assistance of top passivation.^[^ ^36^, ^37^, ^38^, ^39^, ^40^ ^]^ As known and reported, multi‐LG has relatively a large density of state (DOS) than that of monolayer graphene. Nevertheless, it is surprising that the DOS of multi‐LG contact seems quite small enough to practically modulate the Schottky barrier between ambipolar 2D semiconductor and multi‐LG.^[^ ^39^, ^40^ ^]^ Bandgap could thus be estimated via achieving temperature‐dependent transfer curve characteristics of prepared 2D FETs with such contact. These transport gap results were confirmed by spectral photoelectric probing analysis.^[^ ^41^ ^]^ Hence, this application on multi‐LG‐based barrier modulation might be a great merit that we found useful and scientifically meaningful. We regard that our *E*_g_ approximation method via multi‐LG contact is particularly useful for relatively small bandgap ambipolar semiconductors such as thick BP and MoTe_2_,^[^ ^42^, ^43^ ^]^ because measuring their bandgap by any means is substantially difficult.

## 2 Result and Discussion

**Figure 1:**
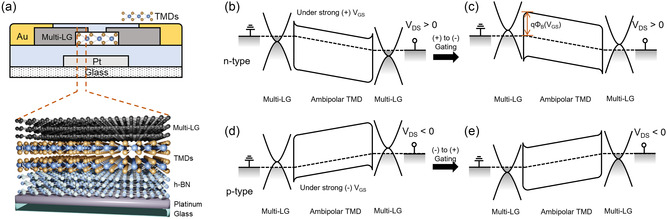
a) Schematic cross section of 2D semiconductor channel with multilayer graphene (multi‐LG) contact. b) Band diagram corresponding to the situation under strong (+) gate voltage (*V*_GS_), which makes the n‐channel. c) Band diagram of neutral intrinsic semiconductor mode, where the Schottky barrier height *q*Φ_B_ becomes maximum. Positive *V*_GS_ is reduced toward (−) *V*_GS_. d) Band diagram corresponding to the situation under strong (−) *V*_GS_, which makes the p‐channel. e) Band diagram of neutral intrinsic semiconductor mode, where the Schottky barrier height *q*Φ_B_ becomes maximum. Negative *V*_GS_ is reduced toward (+) *V*_GS_. It should be noted that multi‐LG/channel *q*Φ_B_ is tuned by *V*_GS_ due to the semiconducting property of multi‐LG.

**Figure** **1**a shows a schematic device cross section of 2D‐layered vdW channel FET with multi‐LG contact, while an essential part of the cross section including top multi‐LG/vdW channel/hexagonal boron nitride (h‐BN) dielectric/Pt gate electrode is also magnified into 3D scheme as indicated by dashed lines. Figure 1b,c describes the schematic band diagrams of n‐FET channel with multi‐LG contact under a positive drain/source voltage (*V*_DS_). When gate voltage (*V*_GS_) is strong positive, the diagram should be described as Figure 1b showing n‐type electron conduction, while diminished positive gate biasing toward negative *V*_GS_ changes the diagram to be depleted n‐channel and eventually to be Figure 1c, a maximum depletion with highest Schottky barrier, which is in fact a half of authentic *E*_g_. (Of course, further negative biasing over transition *V*_GS_ would bring forth type inversion.) It is because multi‐LG would be under the influence of gate voltage to properly modulate the barrier between channel and multi‐LG contact. Such barrier or Fermi‐level (*E*_F_) modulation (or charge modulation) is absolutely undoubted via monolayer graphene (1LG) with small DOS. However, it has also been suspected if multi‐LG can modulate the charges in a degree, because the multi‐LG‐induced modulation with a large DOS is less effective than 1LG‐induced modulation. If multi‐LG contact truly modulates its own *E*_F_ via *V*_GS_ and can resultantly tune the Schottky barrier (*q*Φ_B_),^[^ ^39^, ^40^ ^]^ it will provide a great benefit for easy measurement of *E*_g_ of ambipolar 2D‐layered semiconductor channels. Top contact monolayer graphene would be so difficult to fabricate on 2D‐layered semiconductors, while top multi‐LG contacting is much easier. [Here, it is worth noting that the Fermi level of graphene S/D are always aligned with 2D TMD channel at their van der Waals interface contact in thermal equilibrium.]^[^ ^38^ ^]^ Likewise, when *V*_GS_ is initially negative, the diagram should be described as Figure 1d showing p‐type hole conduction, but reduced negative gate biasing toward positive *V*_GS_ changes the diagram to be depleted p‐channel and eventually to be Figure 1e. As a matter of fact, the Schottky barrier between multilayer 2D channel and multi‐LG is *V*_GS_ dependent or electric (E) field dependent.^[^ ^44^, ^45^ ^]^ Such *V*_GS_ dependence of Schottky barrier can be expressed by temperature‐dependent transfer characteristics (drain current–gate voltage: *I*_D_–*V*_GS_), so that the bandgap of 2D channel may finally be extracted.^[^ ^46^ ^]^

**Figure 2:**
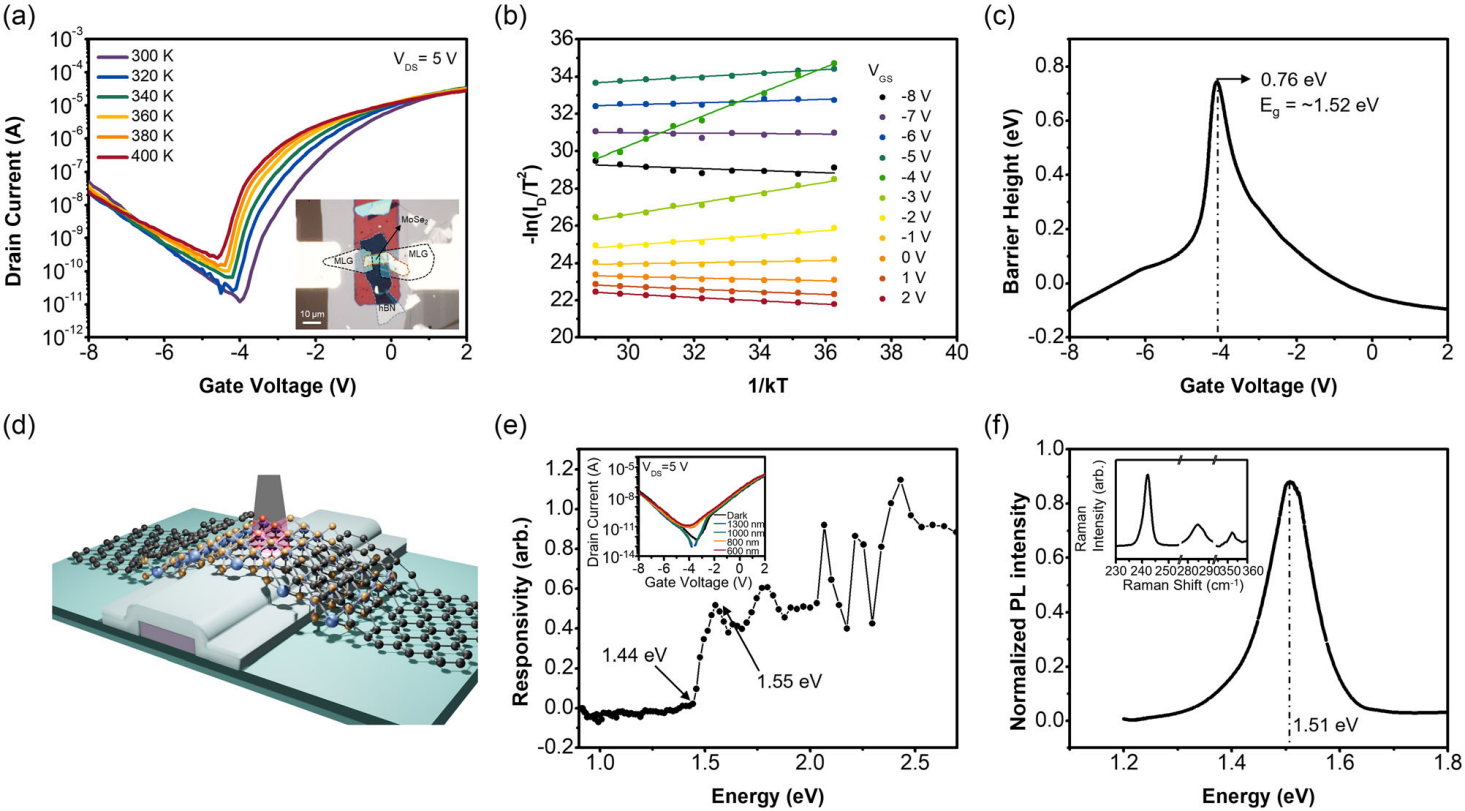
a) Temperature‐dependent transfer characteristics of MoSe_2_ bottom‐gate field‐effect transistor (FET) with multi‐LG contact for source/drain (S/D) whose lead metal is Au. OM image is inset, where thin hexagonal boron nitride (h‐BN) was used for gate dielectric and top passivation. The gate electrode was Pt on glass substrate. b) Arrhenius [−ln(*I*_DS_/*T* ^2^) vs 1/*kT*] plots for each *V*_GS_ condition. c) *V*_GS_‐dependent Schottky barrier height profile extracted from the slope of Arrhenius plots, indicating the peak value and *E*_g_ to be 0.76 and ≈1.52 eV, respectively. d) Optically probed device scheme for photoelectric and photoluminescence (PL) measurements. e) Spectral responsivity plot obtained by photoelectric measurements which come from inset photoinduced transfer *I*–*V* characteristics. These measurements use monochromatic photon beams on the FET in the range from low to high energy and show the onset‐to‐1st peak location between 1.44 and 1.55 eV as a result. f) PL spectra showing the 1.51 eV peak, and inset Raman spectra of the MoSe_2_ channel.

**Figure** **2**a displays the temperature‐dependent transfer characteristics of MoSe_2_ bottom‐gate FET with multi‐LG contact for source/drain (S/D) whose lead metal is Au. An optical microscopy (OM) image is inset, where a thin h‐BN was used for gate dielectric and top passivation, and gate (G) electrode was Pt on glass substrate (see Figure 1a for cross‐section scheme). Thickness information of multi‐LG and h‐BN is provided in Figure S1, Supporting Information, however, MoSe_2_ thickness is unknown and could not be clearly measured by atomic force microscopy (AFM) or any other thickness profiler since it is encapsulated as a channel by other component materials. (So, we initially measured its thickness as shown in the inset AFM profile of Figure S2, Supporting Information, before h‐BN topping. The thickness appears ≈7 nm.) According to the transfer characteristics, the MoSe_2_ FET is an ambipolar device with a negative gate voltage of ≈−4 V as its channel‐type transition point. (Output [drain current–drain voltage: *I*_D_–*V*_DS_] characteristics of the same MoSe_2_ FET is found in Figure S3, Supporting Information, also displaying ambipolar *I*_D_ behavior of decrease and re‐increase at the transition voltage *V*_GS_ ≈−4 V when *V*_GS_ increases from −8 to +2 V.) Gate leakage current, *I*_G_ was very low below ≈pA (not shown here). Through the temperature‐dependent transfer curves in Figure 2a, the Arrhenius [−ln(*I*_DS_/*T* ^2^) vs 1/*kT*] plots of Figure 2b are constructed for each *V*_GS_ condition, and Schottky barrier has been extracted based on Equation (1).^[^ ^40^, ^44^, ^45^, ^46^, ^47^ ^]^

(1) $$I_{\text{DS}}=A^{*}T^{2}\exp\left(-\frac{q\Phi_{B}}{kT}\right)\left[-1+\exp\left(\frac{qV_{\text{DS}}}{kT}\right)\right]$$

where *V*_DS_ is −1 V we use, A* is Richardson's constant, *k* is the Boltzmann constant, and *T* is temperature (Kelvin) and we use *T* ^2^ instead of *T* ^1^ or *T* ^3/2^ because our flake is not a perfect atomistic 2D but ≈10 L thick materials.^[^ ^40^, ^45^, ^47^, ^48^ ^]^ *q*Φ_B_ is Schottky barrier height. We can extract out the *V*_GS_‐dependent barrier heights which are the slopes of ln(*I*_DS_/*T* ^2^) versus 1/*kT* plots at the individual *V*_GS_. Schottky barrier height is varied according to *V*_GS_ as previously explained in Figure 1, and it is plotted in Figure 2c, where the peak value appears to be 0.76 eV at −4.1 V of *V*_GS_. This means that the bandgap of MoSe_2_ channel is close to ≈1.52 eV, twice of 0.76 eV which is the maximum Schottky barrier. At −4.1 V, the MoSe_2_ channel in our ambipolar FET must become an intrinsic semiconductor. This bandgap result and the validity of this measurement technique are interestingly evidenced by two other methods: photoelectric device measurement^[^ ^41^ ^]^ and PL. Figure 2d shows such measurement scheme on a working 2D FET with multi‐LG contact. As shown in Figure 2e and its inset, we achieve photoinduced transfer current–voltage (*I*–*V*) characteristics illuminating monochromatic photon beams on the FET in the range from low to high energy (equipment setup and measurement details are in Figure S4, Supporting Information), and finally plot the responsivity of MoSe_2_ FET. The spectral responsivity plot shows onset‐to‐1st peak location between 1.44 and 1.55 eV. On the one hand, Figure 2f shows the PL result 1.51 eV as an optical gap, as obtained from working MoSe_2_ channel. Hence, our approximation of *E*_g_ is close to the values from optical probing or photoelectric methods, which evidences that the charge modulation of multi‐LG contact is actually effective for this specific use. Inset of Figure 2f is Raman spectra as obtained from working MoSe_2_ channel.

**Figure 3:**
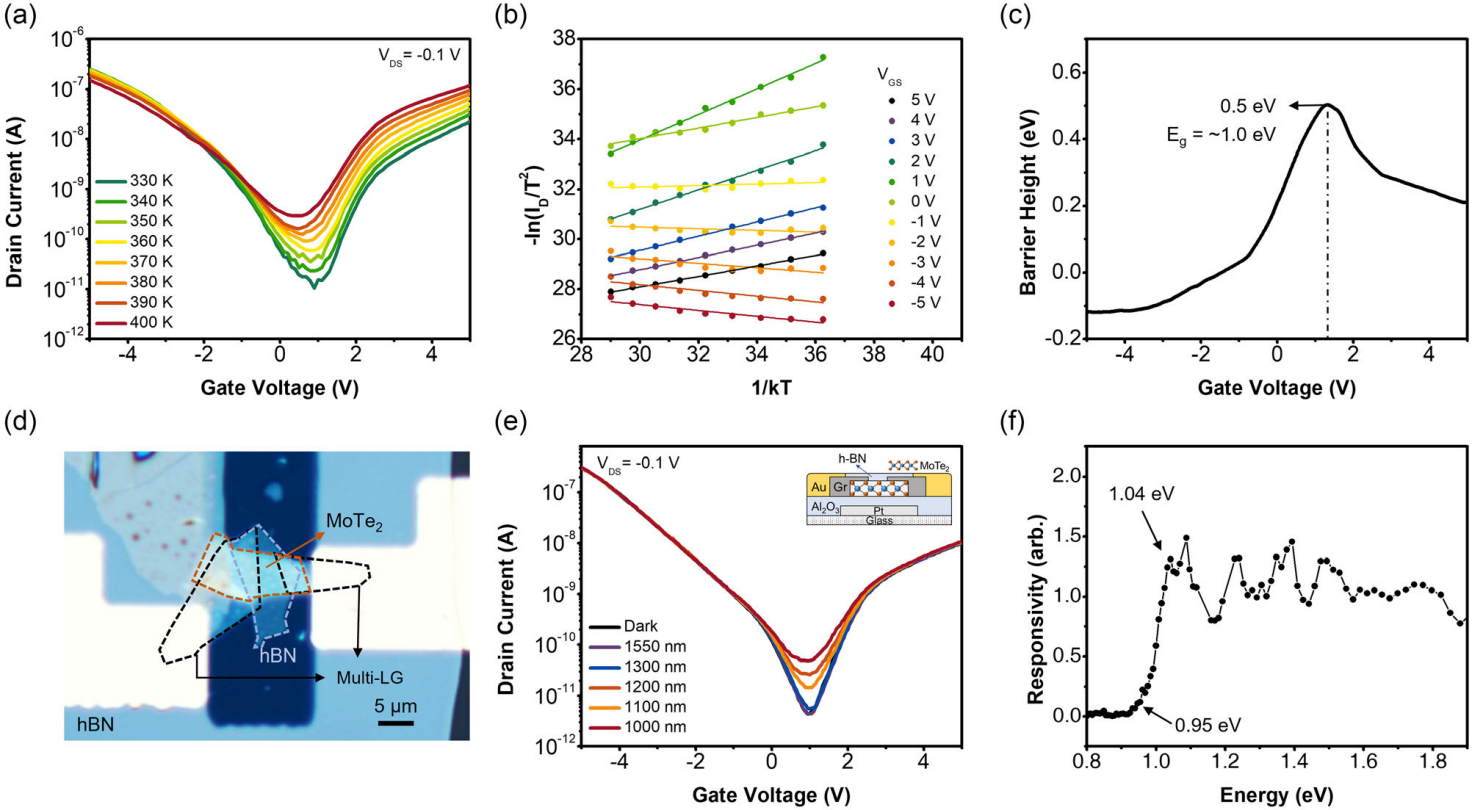
a) Temperature‐dependent transfer characteristics of 4 L MoTe_2_ bottom‐gate FET with multi‐LG contact for S/D whose lead metal is Au. b) Arrhenius [−ln(*I*_DS_/*T* ^2^) vs 1/*kT*] plots for each *V*_GS_ condition c) *V*_GS_‐dependent Schottky barrier height profile extracted from the slope of Arrhenius plots, indicating the peak value and *E*_g_ to be 0.5 and ≈1.0 eV, respectively. d) OM image of the device, where thin h‐BN was used for gate dielectric and top passivation. The gate electrode was Pt on glass substrate. e) Spectral responsivity plot obtained by photoelectric measurements on inset device scheme. f) These measurements brought the *E*_g_ result between 0.95 and 1.04 eV, which is quite consistent with the value (≈1.0 eV) in (c).

Although multilayer MoSe_2_ fortunately shows clear PL results, other ambipolar TMDs such as MoTe_2_ and WSe_2_ are known to show rather unclear or low intensity PL peaks in multilayer and bulk states. (See PL spectra of those in Figure S5, Supporting Information). We now select multilayer MoTe_2_ as an FET channel for the *E*_g_ measurements. **Figure** **3**a–c presents similar fashions to those of Figure 2a–c, as temperature‐dependent transfer curves, Arrhenius plots, and *V*_GS_‐dependent Schottky barrier plot, as achieved from MoTe_2_ FET. The maximum Schottky barrier and approximate *E*_g_ appear to be 0.5 and 1.0 eV near 1.2 V of *V*_GS_, respectively. Because any clear PL spectra of multilayer MoTe_2_ cannot be easy to obtain, we directly put the device to photoelectric *I*–*V* transfer measurements for spectral responsivity. Figure 3d and the inset of Figure 3e are OM image and cross‐section scheme of multilayer MoTe_2_ FET, respectively. According to the results from the photoinduced transfer characteristics in Figure 3e, *E*_g_ of working MoTe_2_ channel is achieved between 0.95 and 1.04 eV, which again proves that our multi‐LG method is valid. We could not directly assess the thickness of MoTe_2_ channel by AFM due to encapsulating top h‐BN, but attempted low‐frequency Raman spectroscopy, where the thickness appears to be ≈4 L (details are in Figure S6, Supporting Information).

**Figure 4:**
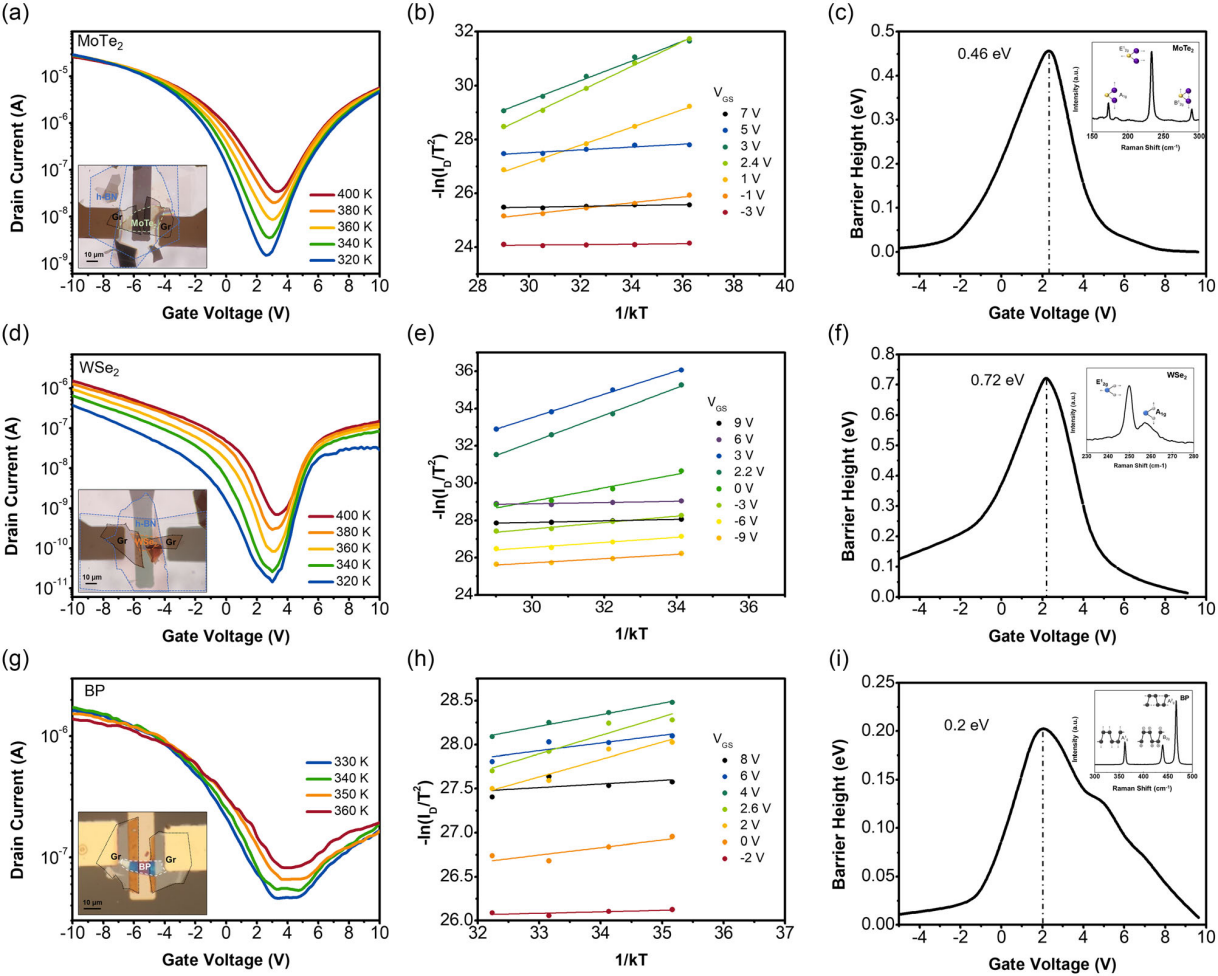
a) Temperature‐dependent transfer characteristics of 9 L MoTe_2_ bottom‐gate FET with multi‐LG contact for S/D whose lead metal is Au. OM image is inset. b) Arrhenius plots for each *V*_GS_ condition. c) *V*_GS_‐dependent Schottky barrier height profile extracted from the slope of Arrhenius plots, indicating the peak value and *E*_g_ to be 0.46 and ≈0.92 eV, respectively. Inset Raman spectra identify MoTe_2_. d) Temperature‐dependent transfer characteristics of 10 L WSe_2_ bottom‐gate FET with multi‐LG contact for S/D. OM image is inset. e) Arrhenius plots for each *V*_GS_ condition and f) *V*_GS_‐dependent Schottky barrier height profile extracted, indicating the peak value and *E*_g_ to be 0.72 and ≈1.44 eV, respectively. The inset Raman spectra identify WSe_2_. g) Temperature‐dependent transfer characteristics of 6 nm black phosphorus (BP) bottom‐gate FET with multi‐LG contact for S/D. OM image is inset with Al_2_O_3_ passivation. h) Arrhenius plots for each *V*_GS_ condition and i) *V*_GS_‐dependent Schottky barrier height profile extracted from the slope of Arrhenius plots, indicating the peak value and *E*_g_ to be 0.2 and ≈0.4 eV, respectively. The inset Raman spectra identify BP.

Since our bandgap estimation results are regarded reasonable, we electrically probed other 2D materials for their bandgaps: another MoTe_2_ (thickness ≈9 L estimated by Raman in Figure S6, Supporting Information), WSe_2_, and BP. **Figure** **4**a and its inset show temperature‐dependent transfer characteristics and OM image of 9 L thick MoTe_2_ channel FET with multi‐LG, respectively. Figure 4b,c display corresponding Arrhenius plots and *V*_GS_‐dependent Schottky barrier plot. A smaller *q*Φ_B_ maximum of 0.46 eV (*E*_g_ ≈ 0.92 eV) is extracted here, which means that this MoTe_2_ must be thicker than that of Figure 3c (4 L thin MoTe_2_ in Figure 3 shows 0.5 eV of *q*Φ_B_ maximum, and 1.0 eV of E_g_). The inset of Figure 4c shows the high‐frequency Raman data to identify the MoTe_2_ through its local vibration modes. For Figure 4d–f, thick WSe_2_ channel FETs are also investigated. Figure 4d and its inset show temperature‐dependent transfer characteristics and OM image of 10 L thick WSe_2_ channel FET with multi‐LG, respectively. The channel thickness was measured with AFM before h‐BN topping. (AFM scan data are shown in Figure S2, Supporting Information, along with additional WSe_2_ channel FET with multi‐LG and 17 L WSe_2_.) Figure 4e,f displays corresponding Arrhenius plots and *V*_GS_‐dependent Schottky barrier plot of 10 L thick WSe_2_ FET. Maximum *q*Φ_B_ of 0.72 eV (*E*_g_ = 1.44 eV) is extracted for 10 L WSe_2_ here, while 17 L WSe_2_ channel appears to show 0.58 eV (*E*_g_ = 1.16 eV, Figure S7, Supporting Information). Finally, 6 nm thick BP channel was taken for our bandgap measurements as shown in Figure 4g and its inset OM image. In this case, we could measure the channel thickness by AFM scan even after top passivation because conformal atomic layer deposition (ALD) of Al_2_O_3_ was conducted against BP oxidation.^[^ ^49^ ^]^ The effective thickness of BP channel was thus measured as seen in Figure S8, Supporting Information, where BP device cross section and Raman spectra of our BP channel are also shown. The maximum Schottky barrier height is extracted to be 0.20 eV from the plots of Figure 4h,i. Therefore, the bandgap of the present BP channel is determined to be 0.40 eV, which is quite comparable to the value in the literature.^[^ ^11^, ^26^ ^]^ Our results on the BP bandgap would not be changed by any doping in the material; however, if any type of surface oxidation was developed and changed/decreased the effective thickness of BP channel, our results might show a little higher bandgap than expected.^[^ ^42^ ^]^ **Table** **1** summarizes the bandgap values of the presented four types of 2D‐layered materials, which are dependent on thickness as cited in literature. According to the table, our experimental approximation of *E*_g_ seems reasonable again in both aspects of 2D materials and their thickness. We thus regard that multi‐LG contact on multilayer vdW 2D‐layered channel can effectively modulate the *E*_F_ and *q*Φ_B_ as a function of *V*_GS_ to approximately obtain the highest *q*Φ_B_ and *E*_g_ of the ambipolar channel semiconductor.

**Table 1:** Summary of the bandgap information on the presented four types of 2D‐layered materials, which are dependent on thickness as cited in literature.^[^ ^4^, ^11^, ^16^, ^20^, ^21^, ^24^, ^50^, ^51^, ^52^ ^]^

2D material	Thickness (layer number)	*E*_g_ (this work) [eV]	*E*_g_ (PL) [eV]	*E*_g_ (photo *I*–*V*) [eV]	*E*_g_ (reference, bulk to 1 L) [eV]
MoSe_2_	7 nm	1.52	1.51	1.44	1.54–1.56^[^ ^51^, ^52^ ^]^
MoTe_2_	4 L	1.0	–	0.99	0.81–1.2^[^ ^16^, ^20^, ^24^ ^]^
MoTe_2_	9 L	0.92	–		
WSe_2_	10 L	1.44	–		1.2–1.64^[^ ^4^, ^21^, ^50^ ^]^
WSe_2_	17 L	1.16	–		
BP	6 nm	≈0.4	–		0.3–2.0^[^ ^11^ ^]^

## 3 Conclusion

We have conducted the bandgap measurements of multilayer van der Waals semiconductor, utilizing multi‐LG contact on ambipolar MoSe_2_, MoTe_2_, WSe_2_, and BP channels in FET. Since those multilayer channels show ambipolar behavior with multi‐LG contact which is able to tune Schottky barrier, their maximum Schottky barrier height is approximated through temperature‐dependent transfer curve characteristics. The maximum Schottky barrier between multilayer channel and graphene is extracted near a transition *V*_GS_ point where minimum *I*_D_ is obtained. At the point, Fermi energy of each channel is at its intrinsic level, and the bandgap is achieved as twice of Schottky barrier height. Our bandgap approximation method appears valid, as evidenced by PL and photoelectric *I*–*V* transfer characteristics on MoSe_2_ and MoTe_2_ channels. Our approximation values are regarded very compatible to those in literature, while this new approach doesn't need any specific equipment except electrical probes. We conclude that our bandgap measurement method is both novel and practical as an important scientific tool to probe the bandgap estimation of multilayer indirect 2D semiconductors.

## 4 Experimental Section

4.1

4.1.1

##### Ambipolar FET Device Fabrication

Glass substrate was cleaned with acetone and ethanol using ultrasonicator. A 50 nm thin Pt patterned gate electrode was deposited on the glass substrate through photolithography and DC sputter deposition. h‐BN flake which was mechanically exfoliated by poly(dimethylsiloxane) (PDMS) was transferred on the Pt‐patterned gate as a gate insulator. Then, exfoliated multilayer MoSe_2_, WSe_2_, and MoTe_2_ flakes were transferred on the h‐BN as transistor channel, respectively. In the same way, exfoliated graphene as S/D contact was transferred on the channel flake and here S/D contact regions should have overlap with gate (G) region in consideration of gating effect on graphene. Finally, as a top passivation, another h‐BN layer was transferred on top of device. In the case of BP channel FET, h‐BN was replaced with 50 nm thick Al_2_O_3_ which was deposited by ALD. For measurement, Au lead pattern was deposited on S/D graphene by photolithography and DC sputter deposition.

##### Device and Materials Characterization

Device characteristics were obtained in the dark by using a semiconductor parameter analyzer (4155C Agilent). For temperature‐dependent characteristics, a hot chuck was used in the probe station. PL and Raman spectroscopy were taken with laser source of *λ* = 532 nm. The thickness of h‐BN and graphene was measured by AFM. For optical responsivity measurement, photo‐excited charge collection spectroscopy (PECCS) system was used, which consists of a 500 W Hg (Xe) arc lamp light source, a grating monochromator (covering spectral range: 400–1500 nm), an optical fiber with core diameter 200 μm, and a semiconductor parameter analyzer (HP 4155C, Agilent Technologies). Details on the equipment are explained in Figure S4, Supporting Information.

## Conflict of Interest

The authors declare no conflict of interest.

## Supporting information

Supplementary Material

## Data Availability

The data that support the findings of this study are available from the corresponding author upon reasonable request.
