# Supplementary material for: Measuring the Bandgap of Ambipolar 2D Semiconductors using Multilayer Graphene Contact
Source: Small Sci. 2022 Dec 22;3(2):2200075. doi: 10.1002/smsc.202200075 (PMC11935821; doi:10.1002/smsc.202200075)
Supplement: Supplementary file 1 — Supplementary Material [file SMSC-3-2200075-s001.pdf]

Supporting Information

Measuring the Bandgap of Ambipolar 2D Semiconductors Using Multilayer Graphene Contact

*Sam Park, Sungjae Hong, June Yeong Lim, Sanghyuck Yu, Jungcheol Kim, Hyeonsik Cheong, Seongil Im**

Supporting Figures

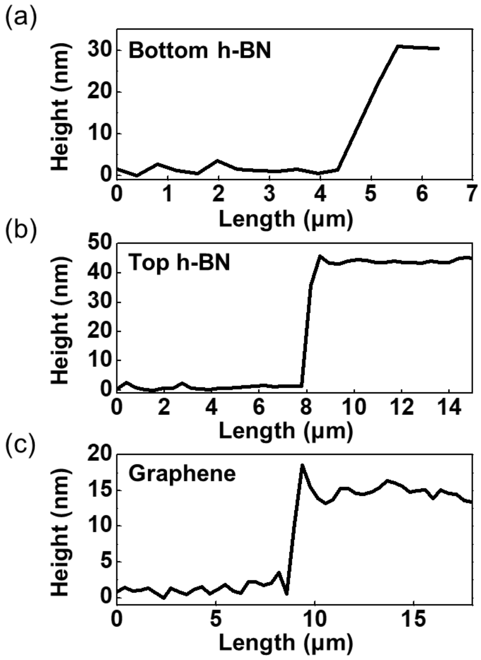

Figure S1. (a) ~ (c) AFM surface topography of (a) bottom h-BN, (b) top h-BN, and (c) multilayer graphene which are used for $MoSe_2$ transistor with multilayer graphene S/D contact.

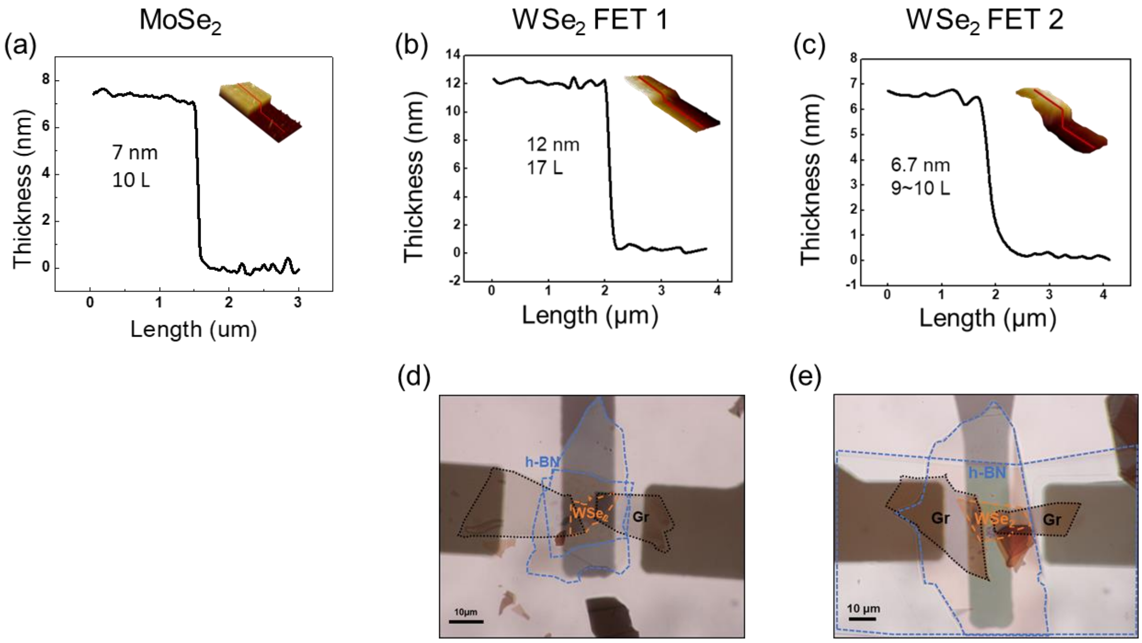

Figure S2. (a) ~ (c) AFM surface topography of (a) $MoSe_2$, (b), (c) WSe_2 flakes which are used for fabrication of transistor with multilayer graphene S/D contact (WSe_2 FET1 in figure S7 and WSe_2 FET2 in figure 4). (d), (e) Optical microscope image of WSe_2 FETs with multilayer graphene S/D contact.

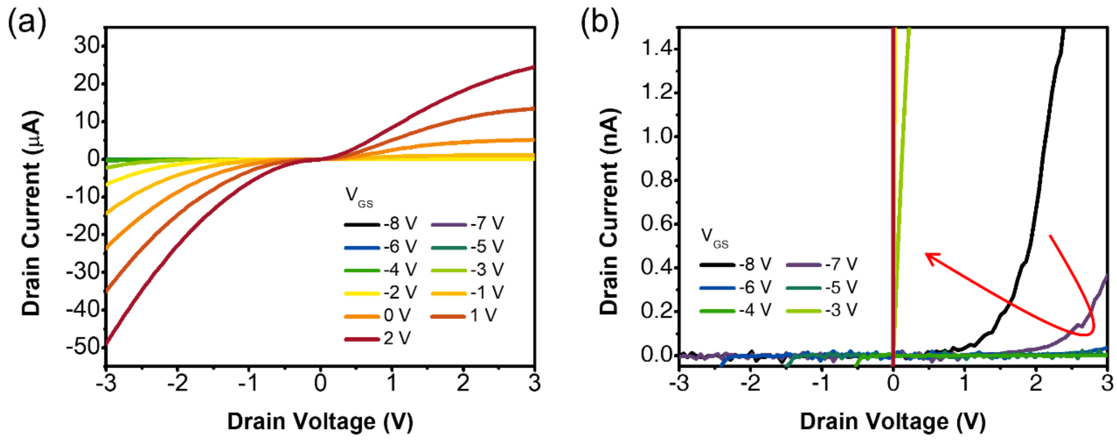

Figure S3. Output characteristics of ambipolar $MoSe_2$ FET. (a) Output curves under gate voltage V_{GS} = -8 ~ 2 V. (b) Magnified output curves to show hole current under negative gate voltage condition. As can be seen in Figure S3(b), drain current re-increase as V_{GS} goes from -4 to -8 V, which indicates the channel type transition from n- to p-type.

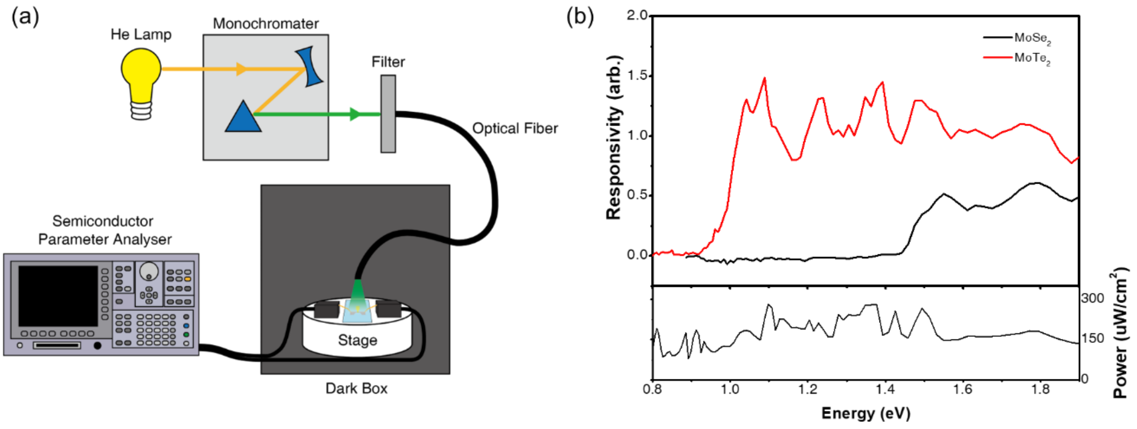

Figure S4. (a) Schematic diagram of photo-responsivity measurement setup which is composed of He lamp, monochromator, filter to get rid of second harmonic component of light, optical fiber to transport the light to device under test, and electrical measurement system. (b) responsivity-photon energy plot (upper) with light power from the light source (lower). Each responsivity is extracted at V_{GS} which minimizes dark current (V_{GS} = -3.6 and 1V for $MoSe_2$ and $MoTe_2$ FET, respectively). The responsivities of $MoSe_2$ and $MoTe_2$ FET are obtained under V_{DS} = 5 and -1 V condition. All the responsivity results are normalized by the light power.

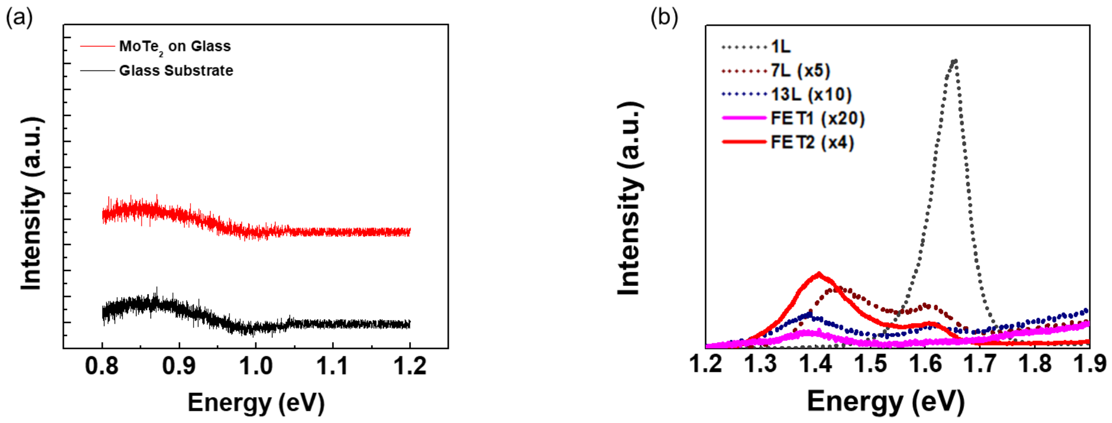

Figure S5. (a) PL spectra of multilayer $MoTe_2$ displayed with PL spectra of bare substrate. A broad peak near 0.85 eV seems from glass substrate, not from $MoTe_2$, because it also appears in PL from bare glass. There is no recognizable peak from $MoTe_2$. (b) PL spectra from 1L, 7L, 13L WSe_2 flakes, and WSe_2 FETs (FET1 and FET2). The bandgap of 1L and 7L WSe_2 appears to be ~1.64 and 1.44 eV which are similar to reported values, and 7L shows two peaks of 1.44 and 1.59 eV as often reported. The PL spectra of WSe_2 FET2 display its main peak at 1.41 eV which is quite close to our result (1.44 eV) from ambipolar FET measurements. Compared with 1L, 7L and 13L, WSe_2 of FET1 is believed even thicker than 13L while WSe_2 in FET2 is as thin as between 7L and 13L. These results imply that our bandgap measurement seems to provide reasonable values (FET1-17L: 1.16 eV, FET2-10L: 1.44 eV), although the band gap of too thick flakes is never easy to estimate by PL measurement.

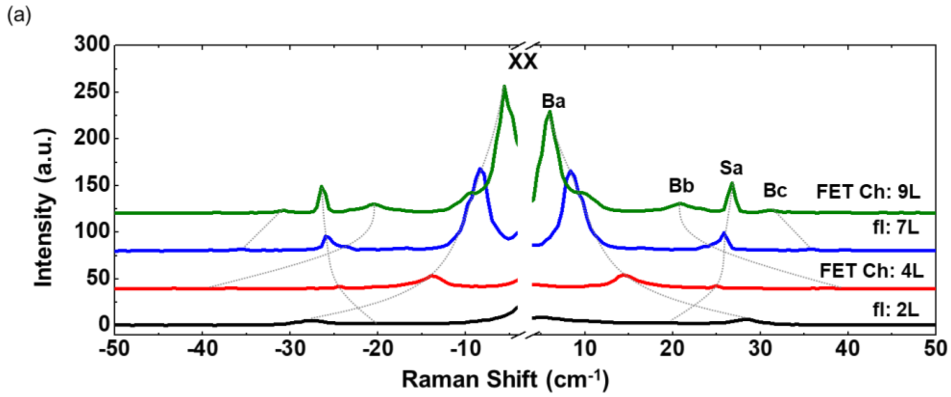

Figure S6. Low frequency Raman spectra of $MoTe_2$ flakes (fl) and our $MoTe_2$ FET channels, recorded at E_L=2.33 eV in the parallel (XX) configuration. By comparing peak positions (Breathing and Shear modes: Ba, Bb, Bc, and Sa) of each flake, our channels turn out to be 4L- and 9L-thick.

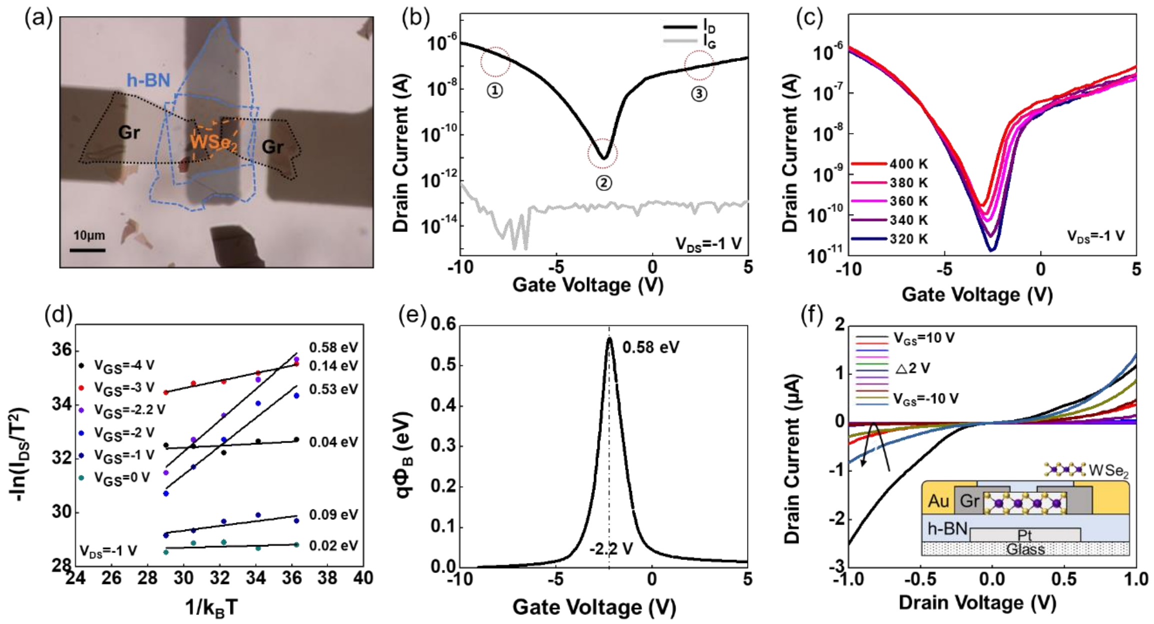

Figure S7. 17L-WSe_2 FET (FET1) with multilayer graphene S/D contact. (a) Optical microscope image. (b) Transfer characteristics under V_{DS} = -1 V. (c) Temperature-dependent transfer curves under V_{DS} = -1 V. (d) Arrhenius plot in which points are extracted from (c). (e) Extracted Schottky barrier height under various gate voltage V_{GS}. (f) Output characteristics of 17L-WSe_2 FET.

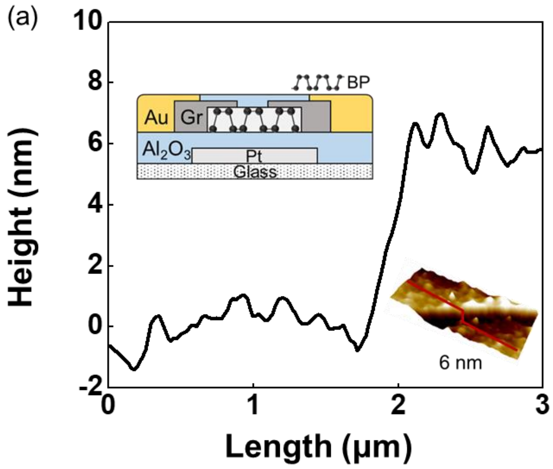

Figure S8. (a) AFM surface line profile, 3D topography (inset, lower-right) and structure (inset, upper-left) of BP flake which is used for BP FET shown in Figure 4(g) ~ (i). The thickness of BP flake is expected to be ~6 nm.
